# Prognostic validation and therapeutic decision‐making of the AJCC eighth pathological prognostic staging for T3N0 breast cancer after mastectomy

**DOI:** 10.1002/ctm2.3

**Published:** 2020-04-23

**Authors:** San‐Gang Wu, Jun Wang, Jian Lei, Chen‐Lu Lian, Li Hua, Juan Zhou, Zhen‐Yu He

**Affiliations:** ^1^ Department of Radiation Oncology The First Affiliated Hospital of Xiamen University Teaching Hospital of Fujian Medical University Xiamen People's Republic of China; ^2^ Department of Radiation Oncology State Key Laboratory of Oncology in South China Collaborative Innovation Center of Cancer Medicine Sun Yat‐sen University Cancer Center Guangzhou People's Republic of China; ^3^ Department of Obstetrics and Gynecology The First Affiliated Hospital of Xiamen University Teaching Hospital of Fujian Medical University Xiamen People's Republic of China

**Keywords:** breast neoplasms, drug therapy, mastectomy, neoplasm staging, radiotherapy

## Abstract

**Background:**

T3N0 breast cancer might be a distinct clinical and biological entity, with higher heterogeneity and presenting diverse responses to locoregional and systemic therapy. The aim of the current study was to validate the prognostic effect and assess the treatment decision‐making of the American Joint Committee on Cancer (AJCC) eighth pathological prognostic staging in T3N0 breast cancer after mastectomy.

**Methods:**

We retrospectively included 2465 patients with stage T3N0 breast cancer who had undergone mastectomy between 2010 and 2014 using the data from Surveillance, Epidemiology, and End Results program. The primary endpoint of this study was breast cancer–specific survival (BCSS).

**Results:**

Of the entire cohort, 76.0% of patients in the seventh AJCC staging system were restaged to the eighth AJCC pathological prognostic staging system. A total of 1431 (58.1%) and 1175 (47.7%) of them received chemotherapy and postmastectomy radiotherapy (PMRT), respectively. Pathological staging was an independent prognostic factor for BCSS. Using pathological prognostic stage IA as the reference, BCSS gradually became worse with increased hazard ratios. The 5‐years BCSS was 96.9%, 95.5%, 91.1%, 85.6%, and 75.5% in pathological prognostic stage IA, IB, IIA, IIB, and IIIA breast cancers, respectively (*P* < .001). In pathological prognostic stage IA, IB, and IIA breast cancers, the receipt of PMRT or chemotherapy was not correlated with better BCSS. However, PMRT was correlated with better BCSS in pathological prognostic stage IIB disease (*P* = .006), but not in pathological prognostic IIIA disease. Moreover, chemotherapy was correlated with better BCSS in pathological prognostic stage IIIA disease (*P* = .006), but not in pathological prognostic stage IIB disease.

**Conclusions:**

The eighth AJCC pathological prognostic staging system provides more risk stratification of T3N0 breast cancers after mastectomy and might affect individualized decision‐making for chemotherapy and PMRT in this patient subset.

**Abbreviations**
AJCC: American Joint Committee on Cancer
BCSS: breast cancer–specific survival
CI: confidence interval
ER: estrogen receptor
HR: hazard ratio
HER2: human epidermal growth factor receptor 2
IDC: infiltrating ductal carcinoma
NCCN: National Comprehensive Cancer Network
NCDB: National Cancer Database
OS: overall survival
PMRT: postmastectomy radiotherapy
PR: progesterone receptor
SEER: Surveillance, Epidemiology, and End Results

## 1 BACKGROUND

The traditionally anatomical American Joint Committee on Cancer (AJCC) TNM system (T, tumor; N, nodes; M, metastasis) has been widely adopted to predict the outcome and treatment decision‐making of breast cancer worldwide.^1^ Breast cancer is a highly heterogeneous entity with diverse prognoses. Several biological factors including histological grade, human epidermal growth factor receptor 2 (HER2), estrogen receptor (ER), and progesterone receptor (PR) status have been identified and validated for their prognostic and predictive role in breast cancer.^2^ The new eighth AJCC pathological prognostic staging system has integrated these biological factors into the anatomical TNM stages.^1^, ^2^, ^3^, ^4^ The effect of new pathological prognostic stages on survival outcomes has been confirmed by several studies, which showed that the new pathological prognostic stages provide accurate prognostic information compared with the anatomical stages.^5^, ^6^, ^7^, ^8^ However, currently, no study has assessed the effect of the new pathological prognostic staging on prognosis and treatment decision‐making in various anatomical TNM stages. In the breast cancer treatment guidelines from the National Comprehensive Cancer Network (NCCN), the recommendation for systemic therapy and postmastectomy radiotherapy (PMRT) still refers to the tumor size and nodal status.^9^

Breast cancer with tumor size greater than 5 cm and negative nodal status was defined as stage T3N0, and accounts for approximately 0.5‐4% of all breast cancers.^10^, ^11^, ^12^, ^13^, ^14^, ^15^ A secondary data analysis from randomized clinical trials showed that chemotherapy and endocrine therapy were not correlated with better locoregional control and lower risk of distant metastasis (DM).^10^ However, two studies from the National Cancer Database (NCDB) showed better overall survival (OS) with the administration of chemotherapy.^16^, ^17^ There were also conflicting results regarding the role of PMRT in this patient subset.^10^, ^11^, ^18^, ^19^, ^20^, ^21^, ^22^, ^23^, ^24^, ^25^ Therefore, T3N0 breast cancer might be a distinct clinical and biological entity, with higher heterogeneity and presenting diverse responses to locoregional and systemic therapy. However, no studies determining the role of the treatment decision‐making in T3N0 breast cancer have been published. Our study aimed to validate the prognostic effect and assess the decision‐making of treatment using the AJCC eighth pathological prognostic staging in T3N0 breast cancer using the data from the Surveillance, Epidemiology, and End Results (SEER) program.

## 2 MATERIALS AND METHODS

### 2.1 SEER database and study population

We conducted a retrospective analysis including female T3N0 breast cancer patients who underwent mastectomy from 2010 to 2014 using the data from the SEER program, a population‐based national cancer registry including tumor incidence, demographic and tumor characteristics, treatment, and survival for approximately 28% of the U.S. population.^26^ Patients with male breast cancer, de novo stage IV disease, nonpositive pathological diagnosis, those treated with nonbeam external irradiation, and insufficient data were excluded. We analyzed the de‐identified information for patients contained in the SEER database; therefore, the present study was exempted from approval by Institutional Review Board.

We identified the patients’ demographic and clinicopathological data, including age, race/ethnicity, tumor grade, histology, HER2, ER, and PR status. In addition, whether chemotherapy or PMRT was administered was also included in the analysis. The classification of pathological prognostic stages was based on the AJCC eighth edition pathological prognostic staging manual.^1^, ^2^

### 2.2 Statistical analysis

A chi‐squared test was performed to compare the patient demographic and clinicopathological characteristics among treatment arms. Kaplan‐Meier analysis was used to calculate breast cancer–specific survival (BCSS), and the difference in BCSS rates was compared using the log‐rank test. The BCSS was defined as the interval from the diagnosis of breast cancer to the date of death from breast cancer or the follow‐up cutoff. Concordance index (c‐statistic) was then used to investigate the discriminatory ability of pathological prognostic staging system in predicting BCSS. Cox proportional hazards regression models were constructed to assess the indicators that were independently related to BCSS. In addition, a competing risks model was also used to investigate the combined effects of the pathological prognostic staging system on breast cancer–specific mortality. Other causes of death were considered as competing events. *P* values < .05 were indicated statistically significant. All data analyses were performed using Stata/SE version 14 (StataCorp, TX) and IBM SPSS 22.0 (IBM Corp., Armonk, NY).

## 3 RESULTS

### 3.1 Patient characteristics

A total of 2465 patients who underwent a mastectomy and had the required information to determine the prognostic stages were included in this study. Table 1 summarizes the patient characteristics. The study cohort included 383 patients (15.5%) with well‐differentiated disease, 1035 (42.0%) with moderately differentiated disease, and 1047 (42.5%) with poorly and/or undifferentiated breast cancer. With regard to hormone receptor status, 72.5%, 60.0%, and 16.0% of the tumors were ER positive, PR positive, and HER2 overexpressed, respectively.

**TABLE 1:** Patient characteristics in the study cohort

		Radiotherapy			Chemotherapy		
Variables	n	No (%)	Yes (%)	*P*	No (%)	Yes (%)	*P*
Age (years)							
<50	652	288 (22.3)	364 (31.0)	<.001	114 (11.0)	538 (37.6)	<.001
≥50	1813	1002 (77.7)	811 (69.0)		920 (89.0)	893 (62.4)	
Race/ethnicity							
White	1889	999 (77.4)	890 (75.7)	.581	831 (80.4)	1058(73.9)	<.001
Black	350	175 (13.6)	175 (14.9)		117 (11.3)	233 (16.3)	
Other	226	116 (9.0)	110 (9.4)		86 (8.3)	140 (9.8)	
Grade							
Well differentiated	383	223 (17.3)	160 (13.6)	.030	239 (23.1)	144 (10.1)	<.001
Moderately differentiated	1035	521 (40.4)	514 (43.7)		506 (48.9)	529 (37.0)	
Poorly/undifferentiated	1047	546 (42.3)	501 (42.6)		289 (27.9)	758 (53.0)	
Histological subtypes							
Infiltrating ductal carcinoma	1503	823 (63.8)	680 (57.9)	<.001	509 (49.2)	994(69.5)	<.001
Infiltrating lobular carcinoma	704	315 (24.4)	389 (33.1)		389 (37.6)	315 (22.0)	
Other	258	152 (11.8)	106 (9.0)		136 (13.2)	122 (8.5)	
ER status							
Negative	677	356 (27.6)	321 (27.3)	.877	167 (16.2)	510 (35.6)	<.001
Positive	1788	934 (72.4)	854 (72.7)		867 (83.8)	921 (64.4)	
PR status							
Negative	986	509 (39.5)	477 (40.6)	.564	292 (28.2)	694 (48.5)	<.001
Positive	1479	781 (60.5)	698 (59.4)		742 (71.8)	737 (51.5)	
HER2 status							
Negative	2071	1075 (83.3)	996 (84.8)	.332	955 (92.4)	1116 (78.0)	<.001
Positive	394	215 (16.7)	179 (15.2)		79 (7.6)	315 (22.0)	
Pathological prognostic stages							
IA	322	194 (15.0)	128 (10.9)	.032	207 (20.0)	115 (8.0)	<.001
IB	890	445 (34.5)	445 (37.9)		431 (41.7)	459 (32.1)	
IIA	228	121 (9.4)	107 (9.1)		96 (9.3)	132 (9.2)	
IIB	591	310 (24.0)	28 1 (23.9)		187 (18.1)	404 (28.8)	
IIIA	434	220 (17.1)	214 (18.2)		113 (10.9)	321 (22.4)	
Chemotherapy							
No	1034	599 (54.2)	335 (28.5)	<.001	–	–	–
Yes	1431	591 (45.8)	840 (71.5)		–	–	–
PMRT							
No	1290	–	–	–	699 (67.6)	591 (41.3)	<.001
Yes	1175	–	–		335 (32.4)	840 (58.7)	

Abbreviations: ER, estrogen receptor; HER2, human epidermal growth factor receptor 2; IDC, infiltrating ductal carcinoma; ILC, infiltrating lobular carcinoma; PMRT, postmastectomy radiotherapy; PR, progesterone receptor.

In the entire cohort, 1431 (58.1%) patients received chemotherapy. Patients with age <50 years, black race, poorly and/or undifferentiated disease, infiltrating ductal carcinoma (IDC), hormone receptor negative disease, and HER2 positive disease were more likely to be treated with chemotherapy (all *P *< .001). In addition, patients with pathological prognostic stage IIB and IIIA breast cancers were also associated with receipt of chemotherapy (Table 1).

A total of 1175 patients (47.7%) had undergone PMRT. Patients with age <50 years, higher tumor grade, non‐IDC histology, and those receiving chemotherapy were more likely to receive PMRT (all *P *< .05). Patients with pathological prognostic stage IIA‐IIIA disease had a comparable probability of receiving PMRT (*P* = .798) (Table 1).

Among the patients with available information on the number of removed lymph nodes (n = 2446), the median number of removed lymph nodes was four (range 1‐42), and 47.8% of them had three or fewer lymph nodes removed.

### 3.2 Restaging

Of the 2465 patients, 76.0% of patients in the seventh AJCC staging system were restaged to the eighth AJCC pathological prognostic staging system, with 17.6% being upstaged and 58.4% downstaged. A total of 322 (13.1%), 890 (36.1%), and 228 (9.2%) patients with seventh edition stage IIB disease were downstaged to pathological prognostic stage IA, IB, and IIA according to the eighth edition criteria. In addition, 434 (17.6%) patients were upstaged to pathological prognostic stage IIIA disease in the eighth edition criteria.

### 3.3 Survival and multivariate prognostic analysis

**FIGURE 1:**
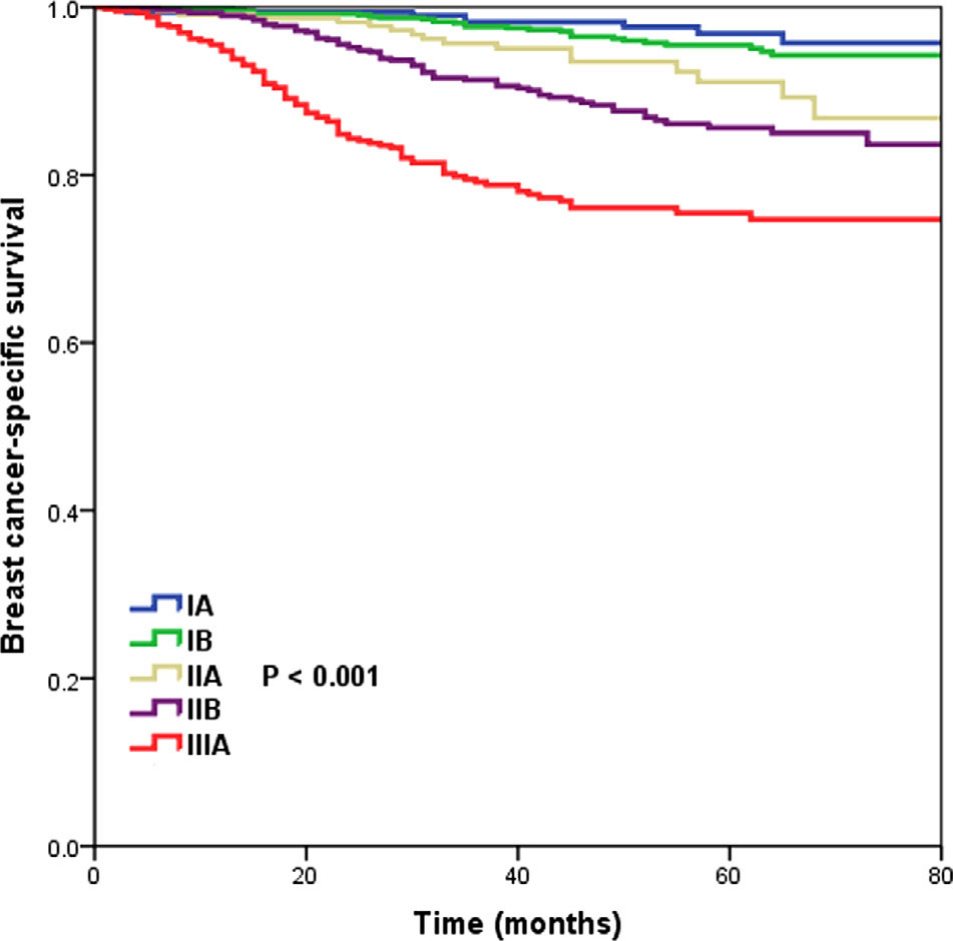
Kaplan‐Meier curves of breast cancer‐specific survival stratified using the eighth AJCC pathological prognostic stages

With a median follow‐up of 45 months (range 0‐83 months), the overall 5‐year BCSS was 89.5%, and was 96.9%, 95.5%, 91.1%, 85.6%, and 75.5% in pathological prognostic stage IA, IB, IIA, IIB, and IIIA breast cancers, respectively (*P* < .001) (Figure 1). However, BCSS was comparable between pathological prognostic stage IA and IB breast cancers (*P* = .272). BCSS was also comparable between pathological prognostic stage IIA and IIB breast cancers (*P* = .063).

The results of multivariate prognostic analysis indicated that pathological prognostic staging was an independent prognostic indicator associated with BCSS. Using pathological prognostic stage IA as the reference, worse BCSS was observed with gradually increasing hazard ratios (HRs). The HR for pathological prognostic stage IIA, IIB, and IIIA disease was 3.082 times (95% confidence interval [CI] 1.307‐7.269, *P* = .010), 5.053 times (95% CI 2.410‐10.596, *P* < .001), and 10.447 times (95% CI 4.981‐21.913, *P* < .001) than that of pathological prognostic stage IA disease, while no significant difference was found between pathological prognostic stage IB and IA disease (HR = 1.668, 95% CI 0.767‐3.630, *P* = .197). In addition, age, histology, and PMRT were also independent prognostic factors affecting BCSS. However, chemotherapy had no effect on BCSS in the entire cohort (Table 2). C‐statistic was assessed using BCSS as the dependent variable, and the pathological prognostic staging demonstrated moderate discriminative ability (*c* = 0.740, SE = 0.016, 95% CI 0.709‐0.771).

**TABLE 2:** Multivariate prognostic analysis in the entire cohort using Cox proportional hazards model and competing risks model

	Cox proportional hazards model			Competing risks model		
Variables	HR	95%CI	*P*	sdHR	95%CI	*P*
Age (continuous variable)	1.022	1.013‐1.032	<.001	1.019	1.007‐1.031	.002
Race/ethnicity						
White	1			1		
Black	1.328	0.954‐1.849	.093	1.319	0.938‐1.854	.111
Other	0.734	0.407‐1.324	.304	0.728	0.407‐1.303	.285
Histological subtypes						
IDC	1			1		
ILC	0.658	0.422‐1.026	.065	0.691	0.449‐1.062	.092
Other	1.440	1.00‐2.055	.045	1.46	0.998‐2.138	.051
Pathological prognostic stages						
IA	1			1		
IB	1.668	0.767‐3.630	.197	1.631	0.750‐3.546	.217
IIA	3.082	1.307‐7.269	.010	3.000	1.290‐6.978	.011
IIB	5.053	2.410‐10.596	<.001	4.817	2.313‐10.030	<.001
IIIA	10.447	4.981‐21.913	<.001	8.984	4.280‐18.859	<.001
Chemotherapy						
No	1			1		
Yes	0.976	0.688‐1.386	.894	1.012	0.701‐1.461	.949
PMRT						
No	1			1		
Yes	0.640	0.481‐0.852	.002	0.656	0.489‐0.881	.005

Abbreviations: CI, confidence interval; HR, hazard ratio; IDC, infiltrating ductal carcinoma; ILC, infiltrating lobular carcinoma; PMRT, postmastectomy radiotherapy, sdHR, subdistribution hazard ratio.

**FIGURE 2:**
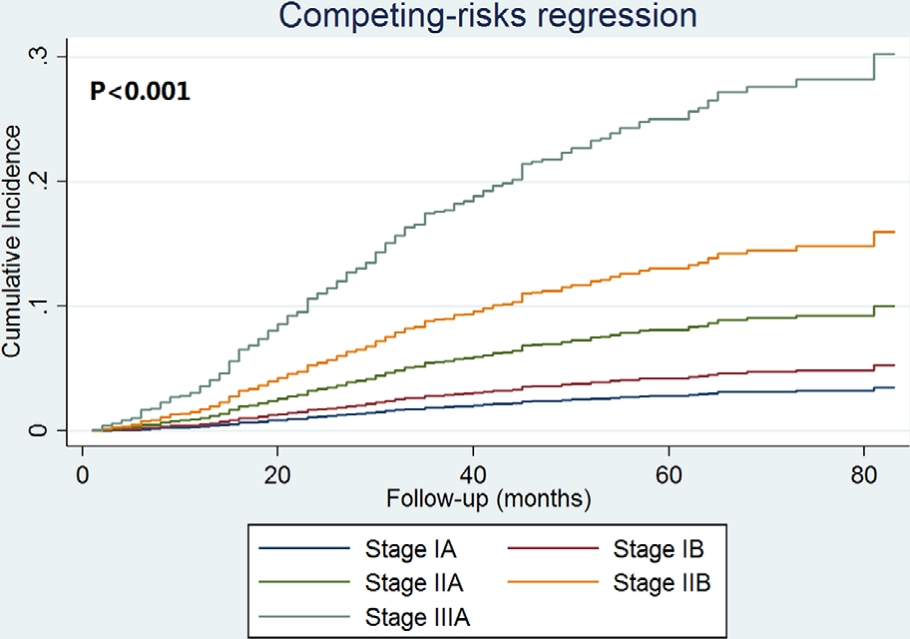
The cumulative incidence function according to the eighth AJCC pathological prognostic stages

Subdistribution hazard ratio (sdHR) adjusted for age at diagnosis, race/ethnicity, histology, chemotherapy, and PMRT was evaluated (Table 2). The results showed an increasing risk of breast cancer–specific mortality with increasing staging. Using pathological prognostic stage IA as the reference, the sdHR for pathological prognostic stage IIA, IIB, and IIIA disease was 3.000 times (95% CI 1.290‐6.978, *P* = .011), 4.817 times (95% CI 2.313‐10.030, *P* < .001), and 8.984 times (95% CI 4.280‐18.859, *P* < .001) compared to pathological prognostic stage IA, while no significant difference was found between pathological prognostic stage IB and IA disease (sdHR = 1.631, 95% CI 0.750‐3.546, *P* = .217). The cumulative incidence function of the pathological prognostic stages is displayed in Figure 2. The 5‐year breast cancer–specific mortality was 3.0%, 4.3%, 8.6%, 13.8%, and 23.3% in pathological prognostic stage IA, IB, IIA, IIB, and IIIA breast cancers, respectively (*P* < .001).

**FIGURE 3:**
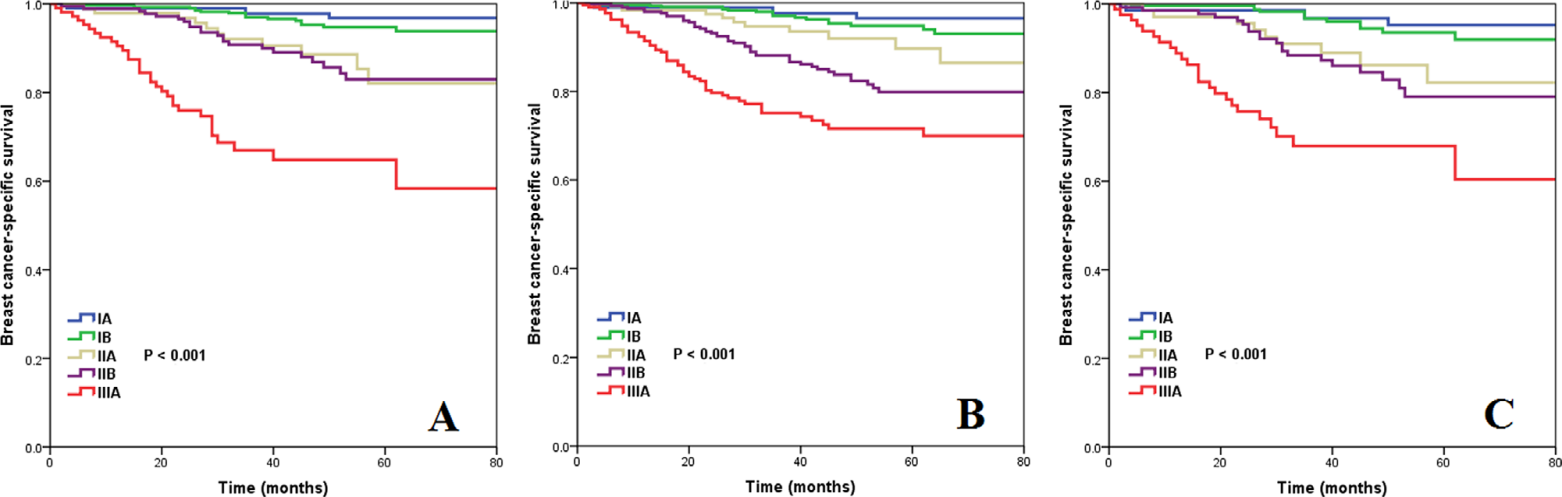
Kaplan‐Meier curves to assess the prognostic effect of pathological prognostic stages on breast cancer‐specific survival by different treatment approaches (A, nonchemotherapy cohort; B, nonradiotherapy cohort; C, nonchemotherapy and nonradiotherapy cohorts)

**FIGURE 4:**
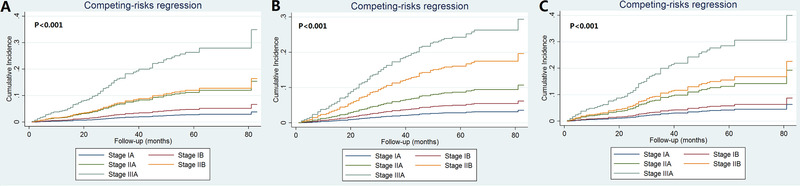
Cumulative incidence function to assess the prognostic effect of pathological prognostic stages on breast cancer‐specific mortality by different treatment approaches (A, nonchemotherapy cohort; B, nonradiotherapy cohort; C, nonchemotherapy and nonradiotherapy cohorts)

We then used three multivariate prognostic models to assess the effect of pathological prognostic staging on BCSS by different treatment approaches (Table 3). The results showed similar results regarding the differences in BCSS among the five pathological substages comprising nonchemotherapy, non‐PMRT, and neither of chemotherapy nor PMRT. In the nonchemotherapy cohort, the 5‐year BCSS was 96.8%, 94.8%, 82.0%, 83.0%, and 64.8% in pathological prognostic stage IA, IB, IIA, IIB, and IIIA breast cancers, respectively (*P* < .001) (Figure 3A); in the non‐PMRT cohort, the 5‐year BCSS was 96.5%, 94.8%, 89.7%, 79.9%, and 71.6%, respectively (*P* < .001) (Figure 3B); and was 95.2%, 93.5%, 82.3%, 79.1%, and 67.9%, respectively, in the nonchemotherapy and non‐PMRT cohort (*P* <0.001) (Figure 3C). However, BCSS was comparable between pathological prognostic stage IA and IB disease in the nonchemotherapy cohort (*P* = .205) and in the nonchemotherapy and non‐PMRT cohort (*P* = .395). In addition, BCSS was also comparable between pathological prognostic stage IIA and IIB disease in the non‐chemotherapy cohort (*P* = .865) and in the non‐chemotherapy and non‐PMRT cohort (*P* = .621). Similar trends were found using cumulative incidence function. The cumulative incidence function of the pathological prognostic stages by different treatment approaches is displayed in Figure 4.

**TABLE 3:** Multivariate prognostic analysis assessing the prognostic effect of pathological prognostic staging by different treatment approaches using Cox proportional hazards model and competing risks model

Variables	Cox proportional hazards model			Competing risks model		
	HR	95%CI	*P*	sdHR	95%CI	*P*
No chemotherapy cohort						
IA	1			1		
IB	1.859	0.689‐5.014	.221	1.755	0.658‐4.683	.261
IIA	4.934	1.712‐14.216	.003	4.240	1.491‐12.061	.007
IIB	4.838	1.827‐12.810	.002	4.547	1.735‐11.919	.002
IIIA	15.107	5.791‐39.407	<.001	10.876	3.968‐29.810	<.001
No PMRT cohort						
IA	1			1		
IB	1.763	0.65‐4.737	.261	1.748	0.654‐4.667	.265
IIA	3.099	1.049‐9.158	.041	3.084	1.070‐8.886	.037
IIB	6.139	2.424‐15.550	<.001	5.957	2.391‐14.840	<.001
IIIA	10.802	4.257‐27.407	<.001	9.463	3.707‐24.158	<.001
Nonchemotherapy and non‐PMRT cohort						
IA	1			1		
IB	1.481	0.528‐4.157	.456	1.396	0.501‐3.891	.524
IIA	3.810	1.276‐11.374	.017	3.275	1.133‐9.473	.029
IIB	4.186	1.567‐11.181	.004	3.922	1.492‐10.310	.006
IIIA	10.678	4.007‐28.454	<.001	7.789	2.808‐21.606	<.001

Abbreviations: CI, confidence interval; HR, hazard ratio; PMRT, postmastectomy radiotherapy; sdHR, subdistribution hazard ratio.

### 3.4 The effect of chemotherapy and PMRT on BCSS in various pathological stages

**FIGURE 5:**
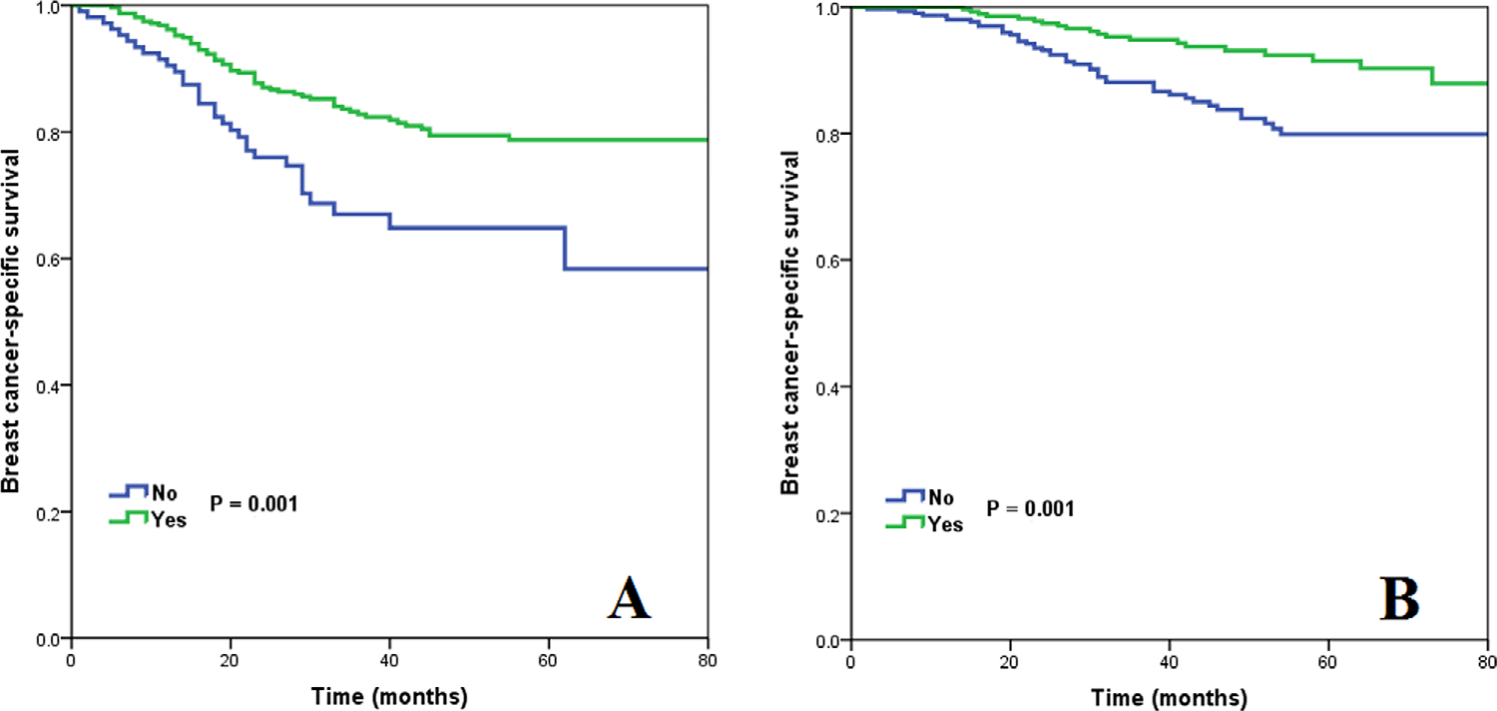
Kaplan‐Meier curves to assess the effect of chemotherapy on breast cancer‐specific survival in patients with pathological prognostic stage IIIA disease (A) and postoperative radiotherapy on breast cancer‐specific survival in patients with pathological prognostic stage IIB disease (B)

**FIGURE 6:**
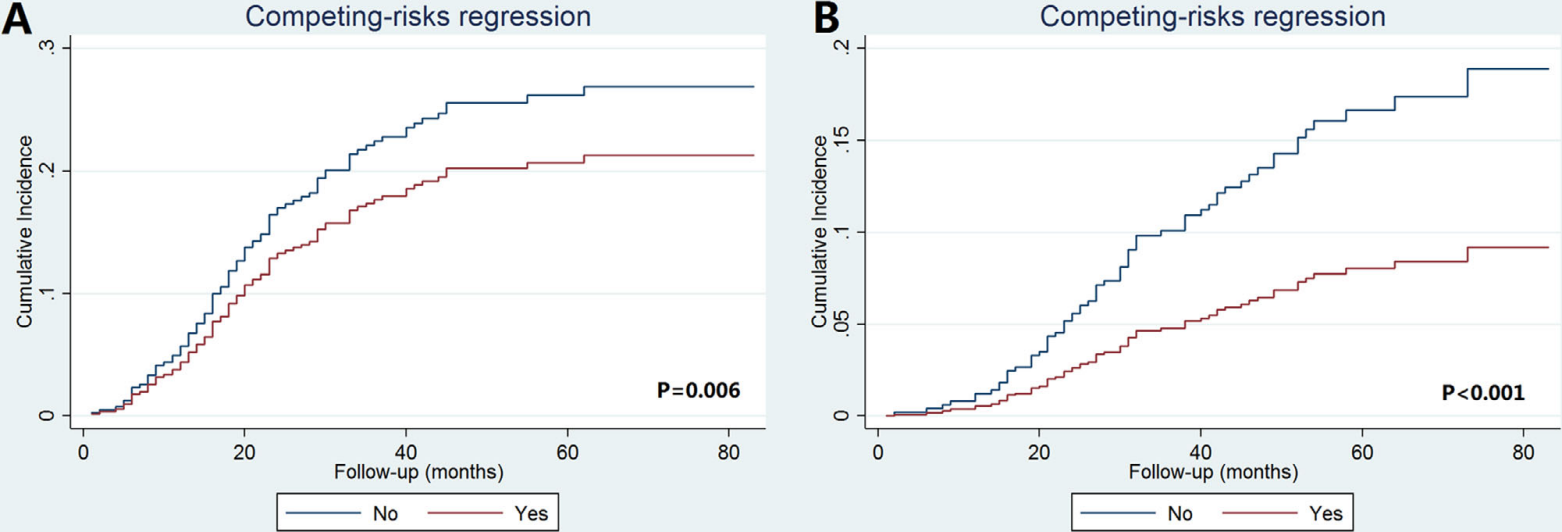
The cumulative incidence function to assess the effect of chemotherapy on breast cancer‐specific mortality in patients with pathological prognostic stage IIIA disease (A) and postoperative radiotherapy on breast cancer‐specific mortality in patients with pathological prognostic stage IIB disease (B)

Five multivariate prognostic models were performed to assess the prognostic effect of chemotherapy and PMRT on BCSS in various pathological prognostic stages after adjustment for age at diagnosis, race/ethnicity, and histology (Table 4). The results showed that the use of chemotherapy or PMRT was not correlated with better BCSS in pathological prognostic stage IA, IB, and IIA breast cancers. However, the use of chemotherapy was related to better BCSS in pathological prognostic stage IIIA disease (HR 0.539, 95% CI 0.347‐0.837, *P* = .006). The 5‐year BCSS was 78.7% and 64.8% in patients with and without chemotherapy (*P* = .001) (Figure 5A), while comparable BCSS was observed between the chemotherapy and nonchemotherapy cohorts in pathological prognostic stage IIB disease. Moreover, the use of PMRT was correlated with better BCSS in pathological prognostic stage IIB disease (HR 0.476, 95% CI 0.279‐0.812, *P* = .006). The 5‐year BCSS was 91.5% and 79.9% in patients with and without PMRT (*P* = .001) (Figure 5B), while comparable BCSS was observed between PMRT and non‐PMRT cohorts in pathological prognostic stage IIIA disease. Similar trends were found using cumulative incidence function. The cumulative incidence function to assess the use of chemotherapy on breast cancer–specific mortality in pathological prognostic stage IIIA breast cancers and PMRT on breast cancer–specific mortality in pathological prognostic stage IIB breast cancers is displayed in Figure 6.

**TABLE 4:** Multivariate prognostic analysis assessing the prognostic indicators associated with breast cancer–specific survival by different pathological prognostic stages

Variables		HR	95%CI	*P*
IA				
Age	Continuous variable	1.065	1.005‐1.128	.032
Race/ethnicity	White	1		
	Black	7.239	1.151‐45.527	.035
	Other			
Histology	IDC	1		
	ILC	1.660	0.270‐10.196	.584
	Other	0.673	0.052‐8.726	.762
Chemotherapy	Yes vs No	3.936	0.594‐26.077	.156
PMRT	Yes vs No	0.710	0.148‐3.410	.668
IB				
Age	Continuous variable	1.044	1.018‐1.071	.001
Race/ethnicity	White	1		
	Black	2.063	0.774‐5.503	.148
	Other	0.740	0.173‐3.160	.684
Histology	IDC	1		
	ILC	1.033	0.481‐2.218	.934
	Other	1.763	0.566‐5.491	.328
Chemotherapy	Yes vs No	1.427	0.617‐3.296	.406
Radiotherapy	Yes vs No	0.751	0.359‐1.570	.447
IIA				
Age	Continuous variable	1.054	1.018‐1.093	.003
Race/ethnicity	White	1		
	Black	2.528	0.644‐9.925	.184
	Other	2.292	0.455‐11.553	.315
Histology	IDC	1		
	ILC	0.197	0.025‐1.540	.122
	Other	2.695	0.328‐22.150	.356
Chemotherapy	Yes vs No	0.436	0.118‐1.607	.212
Radiotherapy	Yes vs No	1.438	0.464‐4.453	.529
IIB				
Age	Continuous variable	1.023	1.005‐1.042	.014
Race/ethnicity	White	1		
	Black	0.981	0.507‐1.901	.956
	Other	0.428	0.133‐1.384	.157
Histology	IDC	1		
	ILC	0.321	0.142‐0.725	.006
	Other	0.482	0.174‐1.336	.160
Chemotherapy	Yes vs No	1.242	0.664‐2.325	.498
Radiotherapy	Yes vs No	0.476	0.279‐0.812	.006
IIIA				
Age	Continuous variable	1.006	0.990‐1.023	.422
Race/ethnicity	White	1		
	Black	1.325	0.842‐2.083	.223
	Other	0.959	0.383‐2.405	.929
Histology	IDC	1		
	ILC	2.064	0.283‐15.070	.475
	Other	2.005	1.289‐3.120	.002
Chemotherapy	Yes vs No	0.539	0.347‐0.837	.006
Radiotherapy	Yes vs No	0.699	0.445‐1.097	.120

Abbreviations: CI, confidence interval; ER, estrogen receptor; HER2, human epidermal growth factor receptor 2; HR, hazard ratio; IDC, infiltrating ductal carcinoma; ILC, infiltrating lobular carcinoma; PMRT, postmastectomy radiotherapy; PR, progesterone receptor.

## 4 DISCUSSION

The present study was the first to validate the prognostic effect of the AJCC eighth edition pathological prognostic staging in T3N0 breast cancer, and to further analyze the impact of treatment decision‐making in different stages. Our results showed that the AJCC eighth edition pathological prognostic staging could better express the risk stratification of patients with T3N0 disease, and that chemotherapy only improved BCSS in pathological prognostic stage IIIA disease, while PMRT was only associated with better BCSS in pathological prognostic stage IIB disease.

After the publication of the eighth AJCC staging system in 2017,^2^ several studies validated the prognostic effect of the new staging compared to the seventh AJCC staging.^5^, ^6^, ^7^, ^8^ However, stage T3N0 breast cancer is a distinct clinical and biological disease,^20^ and there were no studies assessing the newly pathological prognostic stages for this population. Among the 2465 stage T3N0 patients in our study, patients exhibited a worse prognosis with gradually increasing pathological prognostic staging. The 5‐year BCSS was 96.9%, 95.5%, 91.1%, 85.6%, and 75.5% in pathological prognostic stage IA, IB, IIA, IIB, and IIIA breast cancers (*P* < .001). The results indicated that the new pathological prognostic staging system would be the most accurate predictor for risk stratification in stage T3N0 patients. While the anatomical staging system is more straightforward for the classification of T3N0 breast cancer, the newly developed pathological prognostic stages emphasized the equality of tumor burden and biological factors in the era of personalized care.^27^ However, there were comparable BCSS between pathological prognostic stages IA and IB disease, and the results were not influenced by the receipt of systemic therapy and PMRT. In addition, in patients who did not receive chemotherapy, there were also comparable BCSS between pathological prognostic stage IIA and IIB disease. Therefore, further validation studies that included an extended series are required to determine the discriminatory power in predicting survival outcome by pathological prognostic stages.

The new pathological prognostic staging system better reflects the tumor heterogeneity of T3N0 breast cancer and helps to provide more detailed individualized management and prognosis assessment in clinical practice, which suggests that systemic and locoregional management might be escalated or de‐escalated in several anatomical stage groups. However, according to the new NCCN breast cancer treatment guidelines, postoperative chemotherapy is considered for T3N0 breast cancer.^9^ By contrast, neither the European Society for Medical Oncology nor the new St. Gallen International Expert Consensus has a detailed description regarding chemotherapy in T3N0 breast cancer.^28^, ^29^

There is still a lack of recommendations for chemotherapy according to the pathological prognostic staging system. A report from Floyd et al showed that chemotherapy was not correlated with a lower risk of locoregional recurrence (LRR) and DM.^11^ Another secondary data analysis from randomized clinical trials, including 313 patients with T3N0 diseases, found no association of LRR and DM with additional chemotherapy or endocrine therapy.^10^ Although a study from the NCDB showed an OS benefit in the chemotherapy cohort, the use of chemotherapy decreased from 65% in 2004 to 52% in 2012.^16^ Our results also showed that the probability of receiving chemotherapy was higher in pathological prognostic stage IIB and IIIA disease compared to the other three stages, which indicated that biological characteristics were important factors affecting chemotherapy decision‐making in T3N0 breast cancer. We also found that receipt of chemotherapy only improved BCSS in pathological prognostic stage IIIA breast cancer. In the current AJCC staging, pathological prognostic stage IIIA was defined as poorly/undifferentiated and triple‐negative disease with a 5‐year BCSS of only 75.5%. At the recent St. Gallen International Breast Cancer Conference, most of the experts (65.3%) recommended chemotherapy for triple‐negative breast cancer, even in patients with small tumors.^29^ In addition, our results showed that breast cancer‐related mortality in pathological prognostic stage IA and IB breast cancers was extremely low; thus, the survival benefit of systemic chemotherapy was relatively small. However, pathological prognostic stage IIIA cancer was the most high‐risk subgroup, requiring more aggressive adjuvant chemotherapy to improve survival. Moreover, BCSS for pathological prognostic stage IIA and IIB was comparable (5‐year: 82% vs 83%) in nonchemotherapy cohort, suggesting that they may be moderate risk groups, and the best treatment strategy still requires further exploration in pathological prognostic stage IIA‐IIB breast cancers.

The clinical value of PMRT for stage T3N0 breast cancer remains controversial.^20^ Currently, the probability of receiving PMRT varies significantly, and a study from the SEER program showed that 22% of patients were treated with PMRT between 1988 and 1997, and the probability of receiving PMRT increased to 41% from 1998 to 2002.^21^ Another study showed that 42% of patients had received PMRT between 2000 and 2010.^19^ However, a study from the MD Anderson Cancer Center showed that 73.5% of patients received PMRT.^30^ Although the NCCN recommend PMRT in T3N0 breast cancer,^9^ only 47.7% of patients were treated with PMRT in our study. The risk of LRR might have contributed to the decision to recommend PMRT. The results from the Danish Breast Cancer Cooperative Group (DBCG) showed LRR in 17‐23% for this population; however, the median number of removed lymph nodes was only seven, which might affect the accurate assessment of axillary lymph nodes.^24^, ^25^ Two studies with larger cohorts showed lower risks of LRR (7.1‐7.6%) with a higher median removed lymph nodes count was 16.^10^, ^11^ Our previous study showed a median number of removed lymph nodes of 12 and the 5‐years LRR was 6.4%.^31^ In this study, the median number of removed lymph nodes was four, and 47.8% of patients had three or fewer lymph nodes removed. We believe that most of the patients received sentinel lymph node biopsy because the study was carried out in the era of sentinel lymph node biopsy. In the era of modern locoregional and systemic treatment, the risk of LRR in T3N0 breast cancer was lower than previously determined; therefore, PMRT might not be required.^32^

There have been no prospective studies to address the administration of PMRT in T3N0 breast cancer, and to date, most retrospective studies show a questionable benefit of PMRT.^10^, ^18^, ^20^ Two previous study published 20 years ago (DBCG 82b and 82c trials) included T3N0 breast cancers; however, the limited number of enrolled patients make it difficult to draw definitive conclusions regarding the survival benefit of PMRT in this setting.^24^, ^25^ A study from the NCDB included patients diagnosed between 2004 and 2012, among whom PMRT was administered in 45‐50% of patients, and PMRT was related to better OS for this population.^16^ However, several recent retrospective and population‐based studies showed conflicting results.^10^, ^11^, ^18^, ^19^, ^20^, ^21^, ^22^, ^23^, ^24^, ^25^ The study population, clinical pathological features, and differences in treatment patterns might contribute to the significant differences in the above results.

In the present study, the risk of 5‐year BCSS was 96.5%, 94.8%, 89.7%, 79.9%, and 71.6% in the non‐PMRT cohort with pathological prognostic stage IA, IB, IIA, IIB, and IIIA breast cancers, respectively. Although we could not obtain the LRR data from SEER program, in our study, PMRT only improved BCSS in stage IIB breast cancers. Theoretically, there is a positive correlation between the risk of LRR and breast cancer–related death. Patients with low risk of LRR were thought to derive less benefit from PMRT. PMRT had no survival benefit in the high‐risk group, which was similar to the view of Goodman et al.^33^ They showed that a higher risk of LRR might not be converted to survival benefit with additional PMRT.^33^ Moreover, patients in the moderate‐risk cohort might have a lower risk of DM than the high‐risk cohort, and a positive effect of PMRT might be achieved in this cohort. Our findings were similar to the results from the DBCG 82 b&c trials.^34^ Currently, the recommendation of PMRT by the St. Gallen panelists remains controversial (yes vs no, 56.2% vs 43.8%), and 54.3% of the panelists voted for a case‐by‐case decision.^30^ The German expert group applied PMRT only to T3N0 patients with additional risk factors.^35^ However, they did not explain the relevant risk factors. According to our study, the pathological prognostic staging system might be used as a decisive tool to predict the outcome of PMRT in T3N0 breast cancer.

Our study does have limitations. The main limitations are the retrospective nature of this study and selection biases in the nonrandomized dataset. Second, the patterns of LRR and DM are not recorded in the SEER program. Third, treatment information regarding the systemic therapy regimen, endocrine therapy, and anti‐HER2 directed therapy; the sequential use of chemotherapy; and surgery were not captured in the SEER dataset. In addition, the details regarding the target volume, radiation dose, and radiation technology of PMRT are also not included in the SEER program. Finally, the utilization of chemotherapy and radiotherapy in the SEER program was underreported. However, the primary strength of this study is that it represents a large cohort of patients who received the modern era of systemic and locoregional management, and the utilization of a large cohort of T3N0 breast cancer patients provided real world insight that allowed for risk‐stratified analysis of the new pathological prognostic staging system.

## 5 CONCLUSIONS

In conclusion, this study suggests that the newly developed AJCC pathological prognostic stages could provide more risk stratification of T3N0 breast cancer after mastectomy and might affect individualized decision‐making for chemotherapy and PMRT for this population. Further prospective studies are needed to incorporate the newly developed pathological prognostic staging system into the multidisciplinary treatment decision‐making for T3N0 breast cancer.

## AUTHORS’ CONTRIBUTIONS

SGW, JZ, and ZYH are senior authors who contributed in study design. SGW selected patients for the study and collected clinical data. SGW, CLL, JL, and LH performed data analysis, SGW and JW wrote the manuscript. All authors read and approved the final manuscript.

## ETHICAL APPROVAL

We analyzed the de‐identified information for patients contained in the SEER database; therefore, the present study was exempted from approval by the Institutional Review Board.

## CONFLICT OF INTEREST

The authors declare that there is no conflict of interest.

## Data Availability

The data that support the findings of this study are openly available in the Surveillance, Epidemiology, and End Results (SEER) database of the National Cancer Institute at http://seer.cancer.gov/.
